# The association of diastolic arterial pressure and heart rate with mortality in septic shock: a retrospective cohort study

**DOI:** 10.1186/s40001-022-00930-6

**Published:** 2022-12-10

**Authors:** Wenyan Xiao, Wanjun Liu, Jin Zhang, Yu Liu, Tianfeng Hua, Min Yang

**Affiliations:** 1 The 2nd Department of Intensive Care Unit, The Second Affiliated Hospital of Anhui Medical University, Furong Road 678, Anhui, 230601 Hefei People’s Republic of China; 2 The Laboratory of Cardiopulmonary Resuscitation and Critical Care Medicine, The Second Affiliated Hospital of Anhui Medical University, Anhui, 230601 Hefei People’s Republic of China; 3 Research Center for Translational Medicine, the Second Affiliated Hospital of Anhui Medical University, Anhui, 230601 Hefei People’s Republic of China; 4 Key Laboratory of Intelligent Computing and Signal Processing, Anhui University, Ministry of Education, Hefei, Anhui 230601 People’s Republic of China; 5 School of Integrated Circuits, Anhui University, Anhui, 230601 Hefei People’s Republic of China

**Keywords:** Diastolic arterial pressure, Heart rate, Time-dependent intensity, Septic shock

## Abstract

**Background:**

The effects of diastolic arterial pressure (DAP) and heart rate (HR) on the prognosis of patients with septic shock are unclear, and whether these effects persist over time is unknown. We aimed to investigate the relationship between exposure to different intensities of DAP and HR over time and mortality at 28 days in patients with septic shock.

**Methods:**

In this cohort study, we obtained data from the Medical Information Mart for Intensive Care IV, which includes the data of adult patients (≥ 18 years) with septic shock who underwent invasive blood pressure monitoring. We excluded patients who received extracorporeal membrane oxygenation (ECMO) or glucocorticoids within 48 h of ICU admission. The primary outcome was mortality at 28 days. Piece-wise exponential additive mixed models were used to estimate the strength of the associations over time.

**Results:**

In total, 4959 patients were finally included. The median length of stay in the ICU was 3.2 days (IQR: 1.5–7.1 days), and the mortality in the ICU was 12.9%, with a total mortality at 28 days of 15.9%. After adjustment for baseline and time-dependent confounders, both daily time-weighted average (TWA) DAP and HR were associated with increased mortality at 28 days and strong association, mainly in the early to mid-stages of the disease. The results showed that mortality in patients with septic shock was lowest at a DAP of 50–70 mm Hg and an HR of 60–90 beats per minute (bpm). Throughout, a significant increase in the risk of death was found with daily exposure to TWA-DAP ≤ 40 mmHg (hazard ratio 0.99, 95% confidence interval (CI) 0.94–1.03) or TWA-HR ≥ 100 bpm (hazard ratio 1.16, 95% CI 1.1–1.21). Cumulative and interactive effects of harmful exposure (TWA-DAP ≤ 40 mmHg and TWA-HR ≥ 100 bpm) were also observed.

**Conclusion:**

The optimal ranges for DAP and HR in patients with septic shock are 50–70 mmHg and 60–90 bpm, respectively. The cumulative and interactive effects of exposure to low DAP (≤ 40 mmHg) and tachycardia (≥ 100 bpm) were associated with an increased risk of death.

**Supplementary Information:**

The online version contains supplementary material available at 10.1186/s40001-022-00930-6.

## Background

Septic shock is characterized by persistent hypotension and tissue hypoperfusion despite fluid resuscitation [1]. Delays in the diagnosis and initiation of treatment of septic shock are associated with an increased risk of death, and early and effective fluid resuscitation and vasopressor administration are the cornerstones of the treatment of septic shock [2, 3]. Loss of vascular tone is a key pathophysiological feature of septic shock, in addition to relative volume deficiency, and plays a key role in the development of septic shock [1]. A decrease in diastolic arterial pressure (DAP), an important marker of arterial tone, reflects the severity of arteriolar vasodilation [4]. Additionally, low DAP can impair left ventricular myocardial perfusion, especially in tachycardia [5]. Therefore, when DAP is low, such as in early septic shock, there is an increased risk of myocardial ischemia, especially in patients with previous coronary artery disease. Myocardial ischemia can lead to reduced stroke volume and systemic flow, further affecting tissue and microcirculatory perfusion [6].

Although the Surviving Sepsis Campaign (SSC) guidelines recommend achieving a mean arterial pressure (MAP) ≥ 65 mmHg during fluid resuscitation [7–9], intensivists often neglect the importance of DAP and underuse it to manage patients with sepsis despite its simplicity [10]. Importantly, DAP is also not considered in the current SSC guidelines. Its relationship to clinical outcomes has not been extensively described, and no suggestion has been provided on target DAP [10, 11]. However, assessing the loss of vascular tone according to the severity of diastolic hypotension can profoundly impact treatment decisions, such as the early use of norepinephrine. As DAP depends on vascular tone and duration of the cardiac cycle, the combination of DAP and heart rate (HR) can reflect the severity of circulatory dysfunction in the vasodilatory state. Ospina-Tascón et al. showed that gradually opposite changes in HR and DAP show more severe circulatory dysfunction, and the relative risk of death increases proportionally [12]. Moreover, they also found that a diastolic shock index (DSI) (described as HR divided by DAP) > 2.2 was associated with higher mortality in septic shock [12]. Many studies have confirmed that prolonged sinus tachycardia induced by sympathetic excitation is associated with increased major cardiovascular events and higher mortality [13]. However, the effects of DAP and HR on mortality in septic shock have not yet been explored in clinical studies.

Our main objective was to estimate the effect of time-dependent exposure to different intensities of DAP or HR on mortality at 28 days in patients with septic shock. We also examined whether the intensity of the effect varied over time and whether there was a cumulative and interactive effects of exposure time. Finally, we tried to determine the optimal range of DAP and HR.

## Methods

### Study design and participants

We conducted a retrospective cohort study using electronic health record data from the Medical Information Mart for Intensive Care IV (v2.0) (MIMIC-IV 2.0) database, a large open-access database. The MIMIC-IV database contained data of 382,278 patients admitted to the Beth Israel Deaconess Medical Center in Boston, Massachusetts, between 2008 and 2019. Patients meeting the criteria for sepsis after intensive care unit (ICU) admission were eligible for inclusion. Sepsis and septic shock were defined according to the Third International Consensus Definitions for Sepsis and Septic Shock (Sepsis-3) [1]. Sepsis is defined as organ dysfunction caused by infection and is clinically determined based on a sequential organ dysfunction assessment (SOFA) score of ≥ 2 points, which includes patients with confirmed or suspected infection. Septic shock was defined as a MAP < 65 mmHg or vasopressor administration within 24 h after ICU admission. We analyzed only the first stay in the ICU of patients who were admitted to the ICU more than once. Patients who were discharged from the hospital or died within 24 h after admission were excluded. To avoid bias, patients with the following conditions were also excluded: (1) age < 18 years; (2) who received extracorporeal membrane oxygenation (ECMO); (3) MAP ≥ 65 mmHg or did not receive vasopressors within 24 h; (4) received glucocorticoids within 24 h; and (5) did not undergo invasive arterial blood pressure monitoring. The patient selection flow chart is shown in Additional file 1: Fig. S1, Additional file 2. At discharge, all relevant comorbidities were identified using the diagnostic codes of ICD-9 and ICD-10.

### Variable extraction

Data on age, sex, body mass index (BMI), ethnicity, type of admission, and severity at admission were collected using SOFA score, Simplified Acute Physiology Score II (SAPS II), and Oxford Acute Illness Severity Score (OASIS). Comorbidity data were collected to calculate the Charlson comorbidity index (CCI) score. Lifesaving organ support treatment was collected as longitudinal data every 24 h, including continuous renal replacement therapy (CRRT), mechanical ventilation, and vasopressors. If a variable was recorded more than once per day, the worst value associated with the most severe degree of illness was used. Specifically, every 24 h, the time-weighted average (TWA) of the HR or DAP, which is the ratio of the area under the HR or DAP to the timeline, was calculated. In view of the follow-up investigation of MIMIC-IV 2.0 on patients’ outpatient survival, all included patients were followed up from enrollment to 28 days.

### Exposures and outcomes

The primary exposures were the time-dependent TWA-DAP and TWA-HR. Because our study showed a consistent increase in the risk of death with TWA-DAP ≤ 40 mmHg and with TWA-HR ≥ 100 beats per min (bpm) (Additional file 1: Fig. S2), we defined low DAP and tachycardia as TWA-DAP ≤ 40 mmHg and TWA-HR ≥ 100 bpm, respectively. Two methods were used to quantify the effect of harmful exposures: (1) exposure to low DAP or tachycardia during the stay in the ICU and (2) the proportion of time exposure to low DAP and tachycardia.

The primary outcome was mortality at 28 days. Secondary outcomes included ICU mortality and length of ICU stay.

### Statistical analysis

Continuous variables are expressed as mean (standard deviation). Nonparametric variables are expressed as medians (interquartile ranges, IQR). Comparisons between groups were made using the Chi-squared test or Fisher’s exact test for categorical variables and the Mann–Whitney *U* test for continuous variables.

We first applied piece-wise exponential additive mixed models (PAMMs) [14] to estimate the subject-specific longitudinal distributions of TWA-DAP or TWA-HR in relation to mortality at 28 days. PAMMs allow examination of the time-dependent effects of time-dependent exposures (time-dependent TWA-DAP or TWA-HR in this study) on time-to-event outcomes. Based on prior knowledge, baseline variables were purposefully selected as time-dependent confounders in PAMMs, including age, sex, type of admission, BMI, and CCI. We treated longitudinal data during follow-up, including CRRT, vasopressor use, and DAP, as time-dependent confounders in PAMMs. To investigate whether the correlation between TWA-DAP or TWA-HR and mortality at 28 days varied over time, we included an interaction term between time and exposure in the model. We used multiple interlocking equation interpolation to account for the missing data, interpolation was only used in baseline variables (Additional file 1: Fig. S3), and the absence of longitudinal data was allowed in the model.

We determined the approximate DAP and HR thresholds below or above which the risk of 28-day mortality began to increase, based on PAMMs. Three secondary analyses were then performed. We considered any low DAP or tachycardia as a time-dependent exposure variable and investigated the effect of any low DAP or tachycardia exposure on mortality, as well as the relationship between the proportion of harmful exposure time (TWA-DAP ≤ 40 mmHg or TWA-HR ≥ 100 bpm) and mortality. We examined the relationship between cumulative dose and 28-day mortality using areas with longitudinal profiles with TWA-DAP ≤ 40 mmHg or TWA-HR ≥ 100 bpm.

Several subgroup analyses were performed based on the type of admission, gender, comorbidities, and the proportion of exposure time in the ICU (< 10%, 10–30%, 30–50%, 50–70%, > 70%). Furthermore, the interaction and causal mediating effects of tachycardia and low DAP at different exposure times were explored.

Statistical analyses were performed with R (version 4.1). Statistical significance was established at *P* < 0.05.

## Results

Within the MIMIC-IV database, 24,707 patients met the Sepsis 3.0 criteria, and 4959 patients with septic shock were included in this study. The median age of the patients was 67.6 years (IQR: 57.2–77.3); 63.3% were male and predominantly Caucasian. In the study cohort, 71.3% of the patients received vasoactive drugs, 67.9% received invasive mechanical ventilation (IMV), and 2.1% received CRRT. The median stay in the ICU was 3.2 days (IQR: 1.5–7.1), and ICU mortality was 12.9%, with a total mortality of 15.9% at 28 days (Additional file 1: Tables S1, S2). In total, 4959 patients had complete data for TWA-DAP and TWA-HR on day 1, of which 28.7% were exposed to low DAP (TWA-DAP ≤ 40 mmHg) and 66.7% were exposed to tachycardia (TWA-HR ≥ 100 bpm). Patients’ characteristics and outcomes varied widely based on the TWA-HR (Additional file 1: Table S1) and TWA-DAP (Additional file 1: Table S2) levels on day 1.

**Fig. 1:**
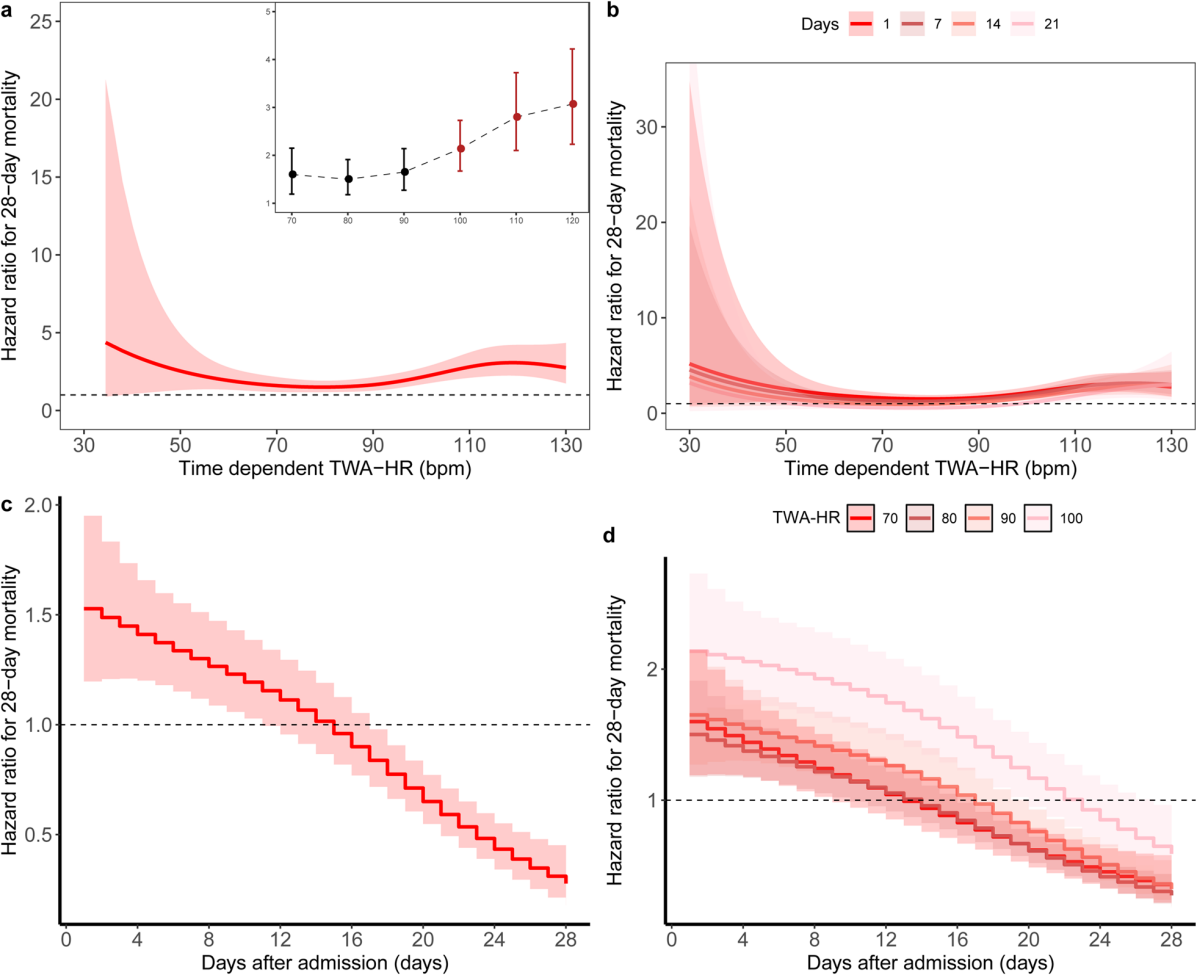
Relationship between daily TWA-HR and 28-day mortality over time using piece-wise exponential additive mixed models. **a** Adjusted relationship between TWA-HR over time and 28-day mortality; **b** Adjusted relationship between TWA-HR over time and 28-day mortality stratified by exposure window; **c** Time-dependent effect of TWA-HR on 28-day mortality; **d** Time-dependent effect of TWA-HR on 28-day mortality stratified by level of HR. *TWA* time-weighted average, *HR* heart rate

**Fig. 2:**
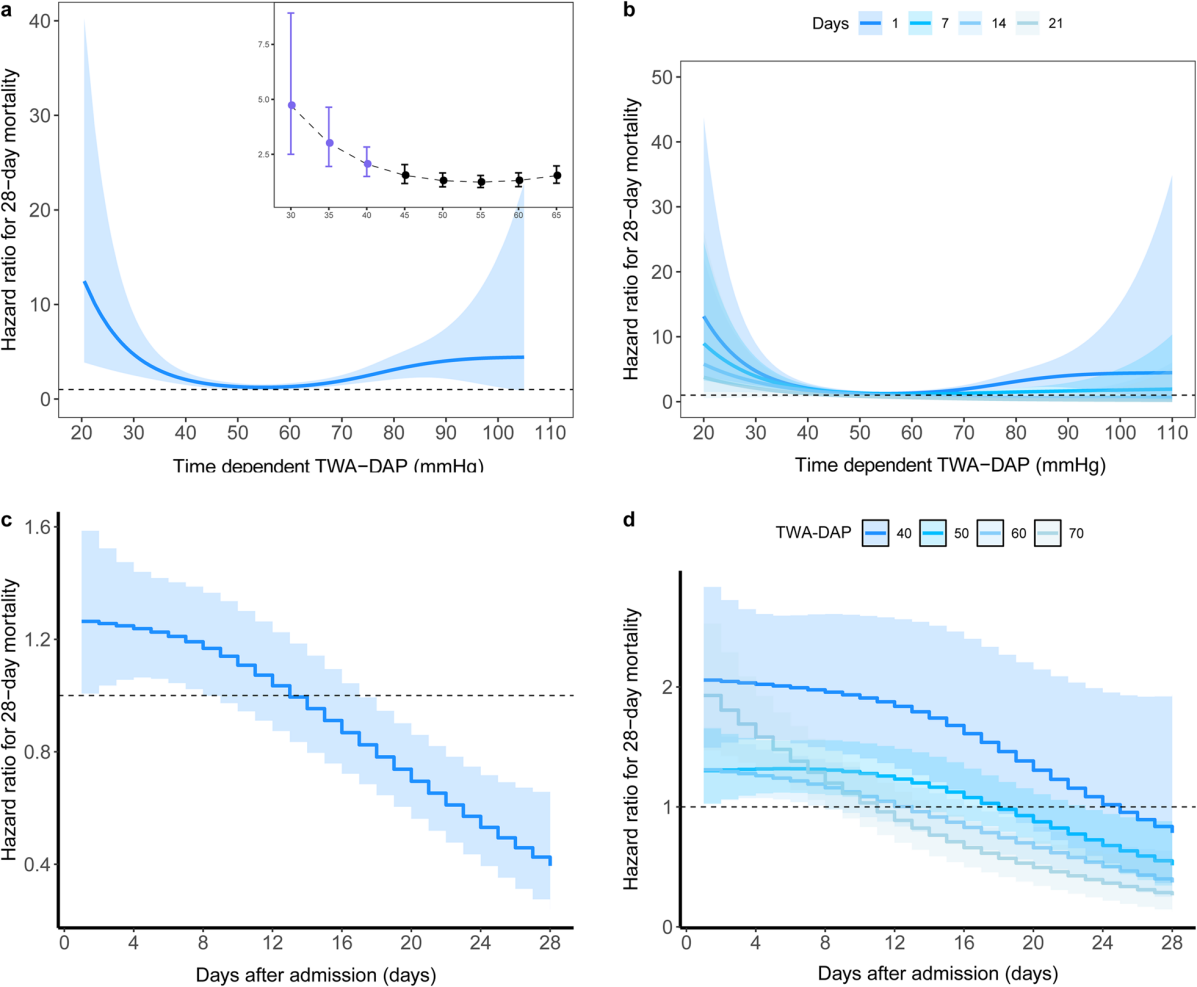
Relationship between daily TWA-DAP and 28-day mortality over time using piece-wise exponential additive mixed models. **a** Adjusted relationship between TWA-DAP over time and 28-day mortality; **b** Adjusted relationship between TWA-DAP over time and 28-day mortality stratified by exposure window; **c** Time-dependent effect of TWA-DAP on 28-day mortality; **d** Time-dependent effect of TWA-DAP on 28-day mortality stratified by level of DAP. *TWA* time-weighted average, *DAP* diastolic arterial pressure

Based on the PAMMs, we determined a U-shaped relationship between time-dependent TWA-HR or TWA-DAP and the risk of 28-day mortality (Figs. 1a and 2a). The results showed that patients with septic shock had the lowest mortality at DAP of 50–70 mmHg (Fig. 1a), while HR of 60–90 bpm (Fig. 2a). The risk of death consistently increased at TWA-HR ≥ 100 bpm (Fig. 1b) and TWA-DAP ≤ 40 mmHg (Fig. 2b). The strength of the correlation between DAP intensity and 28-day mortality, as measured by TWA-HR or TWA-DAP, did not persist during the ICU stay (Figs. 1c, 2c). TWA-DAP had a significant effect on mortality in the early–mid stage of the disease (12–14 days after ICU admission), especially within 7 days (Fig. 2d). Although the effect of TWA-HR was in the early–mid stage of disease (within 14 days after ICU admission) and for higher levels of TWA-HR, its effect on mortality persisted until mid- to late ICU admission (up to 24 days) (Fig. 1d).

After adjusting for age, sex, type of ICU, BMI, CCI, receiving IMV, receiving vasopressors, CRRT, SOFA and lactate, time-dependent TWA-DAP (hazard ratio per 5 mmHg, 0.99 95% CI 0.94–1.03) and TWA-HR (hazard ratio per 10 bpm 1.16, 95% CI 1.1–1.21) were both associated with an increased risk of death at 28 days (Additional file 1: Table S3).

**Fig. 3:**
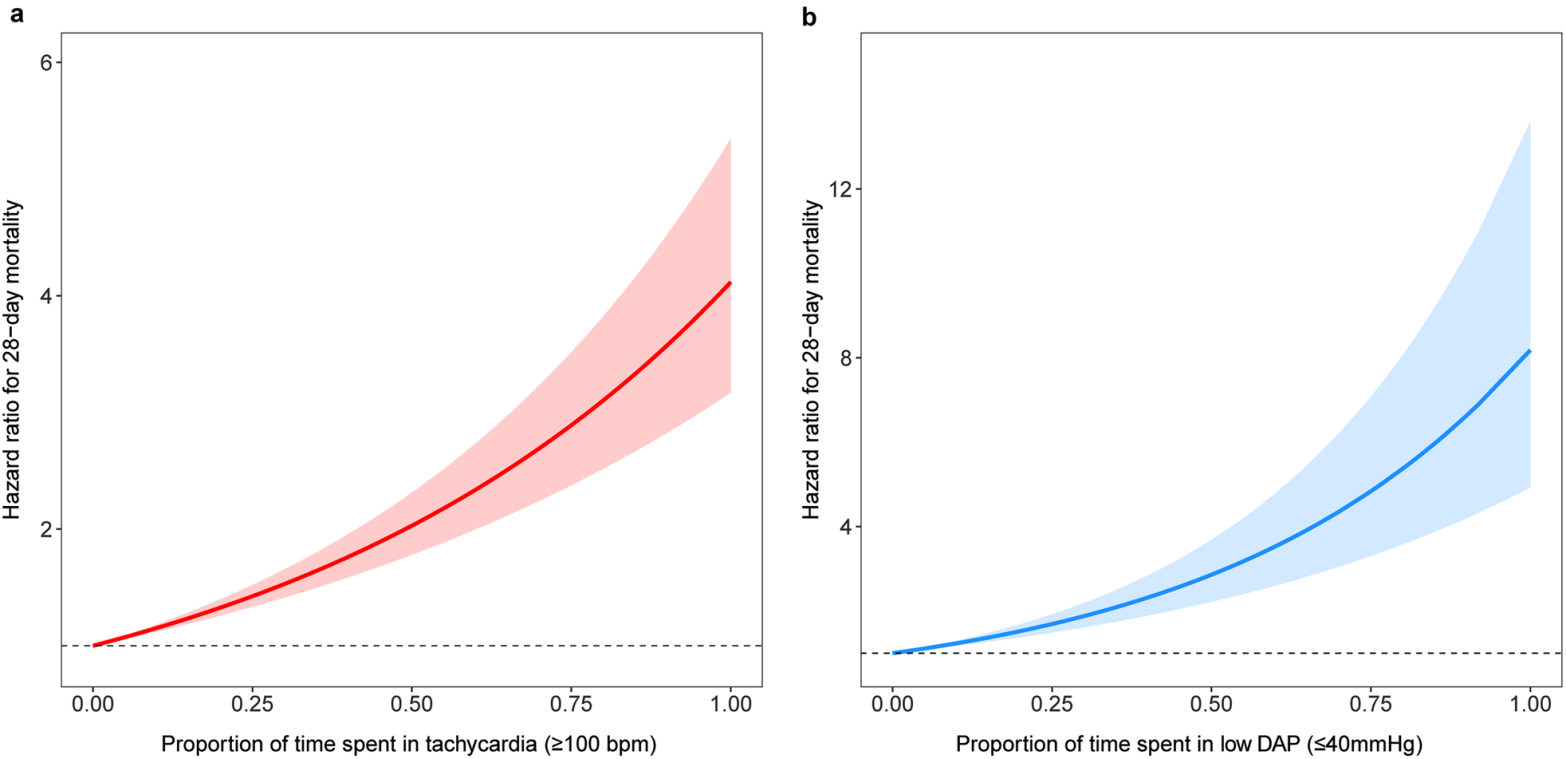
Relationship between proportion of time spent in tachycardia (**a**) or low DAP (**b**) and 28-day mortality using piece-wise exponential additive mixed models

First, exposure to tachycardia (hazard ratio 1.37, 95% CI 1.1–1.7) or low DAP (hazard ratio 1.53, 95% CI 1.31–1.79) was associated with an increased risk of mortality at 28 days (Table 1). Early cumulative mortality due to tachycardia increased rapidly, with cumulative mortality at 28 days reaching approximately 10%. Subsequently, the risk of death was lower in patients without low DAP exposure than in those with overall exposure, while the risk of death was significantly increased in patients with combined low DAP exposure (Additional file 1: Fig. S4). It has been suggested that there may be a potential interaction between tachycardia and low DAP. Secondly, as the proportion of time exposed to tachycardia and low DAP increased, the risk of death increased significantly (Fig. 3), the risk of death caused by tachycardia increased 3.78 times (hazard ratio 3.78, 95% CI 2.91 to − 4.9), and lower DAP leads to a 6.41 times higher risk of death (hazard ratio 6.41, 95% CI 3.86–10.65) (Additional file 1: Table S4). In general, lower DAP was associated with a higher risk of death. Finally, no causal effect was found in the model of the mediating effect of tachycardia and low DAP (Additional file 1: Fig. S5), whereas a sharply increased risk of death was found when patients were exposed to both tachycardia and low DAP, increasing with the proportion of exposure time via the interaction effect (*p* for interaction < 0.001) (Additional file 1: Fig. S6).

**Table 1:** Effects of exposure to tachycardia and low DAP on 28-day mortality in patient with septic shock

	Exposure to tachycardia (HR ≥ 100 bpm)			Exposure to low DAP (DAP ≤ 40 mmHg)		
	HR	95%CI	*P* value	HR	95%CI	*P* value
*Baseline variables*						
Age, years	1.02	(1.01–1.03)	< 0.001	1.01	(1.01–1.02)	< 0.001
Male	0.81	(0.69–0.94)	0.004	0.85	(0.73–0.98)	0.026
BMI, kg/m^2^	0.99	(0.98–1.00)	0.075	0.99	(0.98–1.00)	0.097
CCU	0.33	(0.28–0.40)	< 0.001	0.32	(0.27–0.38)	< 0.001
OASIS	1.02	(1.01–1.03)	< 0.001	1.02	(1.02–1.03)	< 0.001
SOFA	1.09	(1.06–1.11)	< 0.001	1.08	(1.05–1.11)	< 0.001
CCI	1.10	(1.06–1.13)	< 0.001	1.09	(1.06–1.12)	< 0.001
Lactate, mmol/l	1.11	(1.09–1.13)	< 0.001	1.11	(1.09–1.13)	< 0.001
*Time-dependent variables*						
Receiving IMV	1.24	(1.01–1.52)	0.034	1.24	(1.01–1.52)	0.032
Receiving CRRT	1.95	(1.52–2.50)	< 0.001	1.81	(1.41–2.32)	< 0.001
Use of vasopressors	3.20	(2.66–3.87)	< 0.001	3.15	(2.61–3.80)	< 0.001
Tachycardia (≥ 100bmp)	1.37	(1.10–1.70)	< 0.001			
Low DAP (≤ 40 mmHg)				1.53	(1.31–1.79)	< 0.001

*HR* hazard ratio, *CI* confidence interval, *BMI* body mass index, *OASIS* Oxford Acute Severity of Illness Score, *SOFA* sequential organ failure assessment, *CCI* Charlson Comorbidity Index, *CRRT* continuous renal replacement therapy, *IMV* invasive mechanical ventilation, *CCU* Cardiac Care Unit, *TWA* time-weighted average, *DAP* diastolic arterial pressure, *HR* heart rate, *bpm* beats/breaths per minute

**Fig. 4:**
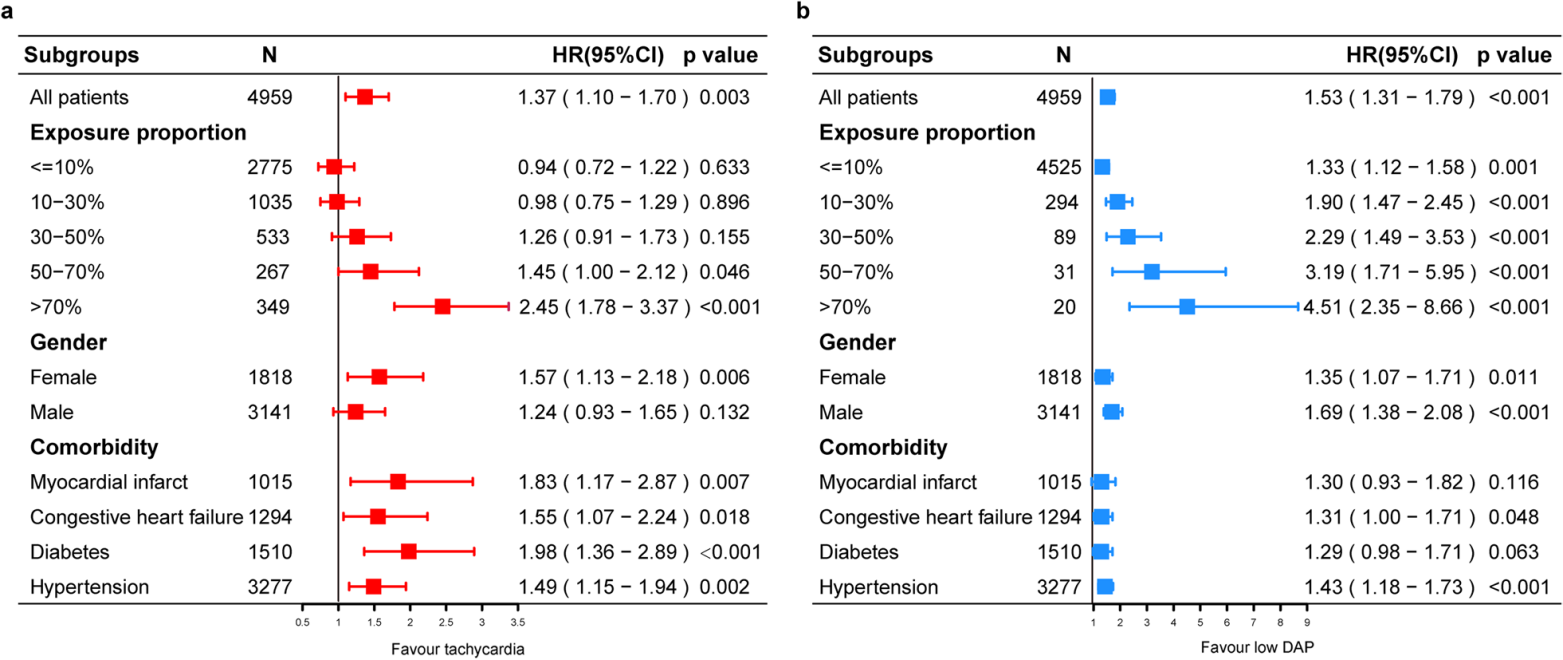
Subgroup analyses of the relationship between different proportions of time exposure to tachycardia (**a**) or ow DAP (**b**) and 28-day mortality over time using piece-wise exponential additive mixed models. *HR*  hazard ratio, *CI*  confidence interval

The subgroup analysis of the relationship between time-dependent low DAP, tachycardia, and 28-day mortality is shown in Fig. 4. As the proportion of exposure time increased, the risk of death from tachycardia increased significantly only after a long time (> 50%), and when the proportion of exposure time was > 70%, the risk of death increased 2.45 times (hazard ratio 2.45, 95% CI 1.78–3.37) (Fig. 4a), while exposure to low DAP increased the risk of death, and when the proportion of exposure time was > 70%, the risk of death increased 4.51 times (hazard ratio 4.51, 95% CI 2.35–8.66) (Fig. 4b). Finally, tachycardia affected the prognosis of patients with myocardial infarction, diabetes mellitus, congestive heart failure, and hypertension and that of women, while low DAP affected the prognosis of patients with hypertension and that of men.

## Discussion

The time-dependent intensity of HR and DAP exposure, measured by daily TWA-HR or TWA-DAP, was associated with increased mortality at 28 days in patients with septic shock, and the strength of this association was predominant in the early-middle course of illness, particularly within 7 days. Patients in septic shock have the lowest mortality at a DAP of 50–70 mmHg and an HR of 60–90 bpm. We also found a cumulative effect of harmful exposure (TWA-DAP ≤ 40 mmHg or TWA-HR ≥ 100 bpm) over time. Moreover, tachycardia and low DAP had an interactive effect on the increased risk of death with prolonged exposure times. Tachycardia and low DAP have different effects on the risk of death in different populations.

With the emergence of a large amount of evidence-based medical evidence, the SSC guidelines have been updated several times [7–9]. Current guidelines emphasize the importance of MAP in sepsis and septic shock, whereas the value of DAP is rarely mentioned [15]. Since loss of vascular tone is a crucial pathophysiological feature of septic shock, the effect of DAP, as a marker of vascular tone, on the clinical outcomes of patients with septic shock has not been elucidated and is easily overlooked by intensivists [6, 11]. Although it is well known that DAP is important for maintaining tissue perfusion [16] and organ function [17, 18], current guidelines do not recommend an appropriate target DAP [6, 10, 11].

From a physiological point of view, DAP is not only a marker of arterial vascular tone but also an upstream pressure of left ventricular perfusion [19]. As long as the aortic valve is normal and the vascular tone from the ascending aorta to the peripheral vessels remains almost constant, detection of DAP in the peripheral vessels can reflect the vascular tone [4]. DAP depends on the vascular tone and duration of the cardiac cycle, since the coronary arteries provide perfusion to the left ventricle only during diastole [16]. Low DAP can impair left ventricle myocardial perfusion, especially in tachycardia with limited diastolic time [20]. Tascón et al. found that DAP and HR simultaneously moving in opposite directions may indicate more severe cardiovascular dysfunction, while isolated DAP or HR values could not clearly identify this risk [12].

Previous retrospective studies exploring the effect of DAP on outcomes in patients with septic shock were limited by a single measurement of DAP, that is, within 24 h after admission to the ICU, and did not account for the interaction between tachycardia and DAP [21, 22]. Biologically, a single exposure can alter the results so dramatically that they are unreliable. Although Tascón et al. proposed that DSI could reflect the prognosis of patients with septic shock, they only compared the change in DSI before and after the use of vasopressors [12]. To resolve this defect, we evaluated exposures to DAP and HR throughout admission to the ICU (up to 28 days) and treated daily DAP and HR as time-dependent variables in PAMMs to explore their effects over time. Second, previous studies believed that DAP < 50 mmHg would affect the prognosis of patients with sepsis, but the grade of clinical evidence was exceptionally low [22]. In this study, we identified thresholds for DAP and HR below (≤ 40 mmHg) or above (≥ 100 bpm), which significantly increased the risk of mortality at 28 days, suggesting that norepinephrine can be used as soon as possible when DAP < 40 mmHg, especially in tachycardia [11]. Third, in previous studies, multivariate regression models were adjusted only for baseline confounders, which may have led to residual confounders [14]. The difference in this study was that we used PAMMs to account for both baseline and time-dependent confounders.

We visualized the longitudinal association between TWA-DAP or TWA-HR and mortality at 28 days throughout ICU admission and suggested that intensivists should pay more attention to DAP and HR during the early to mid-stages of illness, especially within 7 days after ICU admission. The results showed that the optimal range of DAP is 50 to 70 mmHg, while HR is 60 to 90 bpm as much as possible to not affect myocardial perfusion. Assessing cumulative exposure is equally important. For the first time, we found that both time-dependent low exposure to DAP and tachycardia are associated with harm. With prolonged exposure to tachycardia, the effect on mortality progressively increased with time. However, exposure to low DAP was associated with increased mortality, and the interaction between low DAP and tachycardia was more significant. This finding suggests that the incorporation of DAP into the management of septic shock may have profoundly impact treatment decisions, such as the early use of norepinephrine [23]. Furthermore, the effects of exposure infarction, hypertension, and other specific populations on tachycardia and low DAP are different, and the treatments should also be different.

### Limitations

This study has several limitations. First, our study design was a retrospective observational study, and MIMIC-IV is a single-center database; this study has some selection bias, which limits the extrapolation of our conclusions. Second, our diagnostic criteria for septic shock are not completely equivalent to Sepsis-3, making it necessary to further verify the results by clinical studies based on Sepsis-3. Finally, we excluded patients who did not use invasive blood pressure monitoring, because of doubts regarding the accuracy of DAP when obtained using a noninvasive blood pressure device [24]. In fact, the device did not directly measure the DAP. Therefore, for patients with septic shock, we also recommend the insertion of an arterial catheter so that DAP can be measured directly, increasing the reliability of the results [12, 21].

## Conclusions

DAP and HR should be closely monitored in patients with septic shock, especially in the early to mid-stages after ICU admission. The optimal ranges for DAP and HR in patients with septic shock are 50–70 mmHg and 60–90 bpm, respectively. Cumulative exposure to low DAP (≤ 40 mmHg) and tachycardia (≥ 100 bpm) is associated with an increased risk of death, and there are interactive effects between low DAP and tachycardia.

## Supplementary Information

**Additional file 1: Figure S1.** Study flow diagram in the current study. **Figure S2.** Time-dependent effect of TWA-HR (**a**) or TWA-DAP (**b**) on 28-day mortality. **Figure S3.** Distribution of missing values for baseline variables. **Figure S4.** Cumulative effect of tachycardia and low DAP on 28-day mortality. (**a**) Cumulative mortality exposure to tachycardia. (**b**) Cumulative mortality exposure to tachycardia without low DAP. (**c**) Cumulative mortality exposure to tachycardia combined low DAP. **Figure S5.** The direct and indirect effects of tachycardia and low DAP on 28-day mortality. **Figure S6.** The interaction effect of proportion of time spent in tachycardia and low DAP on 28-day mortality.

**Additional file 2: Table S1.** Baseline characteristics and outcomes of patients stratified by TWA-HR on Day 1. **Table S2**. Baseline characteristics and outcomes of patients stratified by TWA-DAP on Day 1. **Table S3** Effects of time-dependent TWA-HR and TWA-DAP on 28-day mortality in patients with septic shock. **Table S4**. Effects of proportion of exposure time in tachycardia and low DAP on 28-day mortality in patients with septic shock.

## Data Availability

The datasets are available in PhysioNet (https://physionet.org/content/mimiciv/2.0/).

## Abbreviations

DAP: Diastolic arterial pressure
HR: Heart rate
MIMIC-IV: Medical Information Mart for Intensive Care IV
TWA: Time-weighted average
MAP: Mean arterial pressure
DSI: Diastolic shock index
ICU: Intensive care unit
ICD: International Classification of Diseases
BMI: Body mass index
SAPS II: Simplified acute physiology score II
OASIS: Oxford Acute Severity of Illness Score
SOFA: Sequential organ failure assessment
CCI: Charlson Comorbidity Index
CRRT: Continuous renal replacement therapy
IMV: Invasive mechanical ventilation
RR: Respiratory rate
MAP: Mean arterial pressure
bpm: Beats/breaths per minute
PAMMs: Piece-wise exponential additive mixed models
MICE: Multiple interlocking equations interpolation

## References

[1] Singer M, Deutschman CS, Seymour CW, Shankar-Hari M, Annane D, Bauer M (2016). The third international consensus definitions for sepsis and septic shock (Sepsis-3). JAMA.

[2] Jozwiak M, Hamzaoui O (2022). Adherence to surviving sepsis campaign guidelines 2016 regarding fluid resuscitation and vasopressors in the initial management of septic shock: the emerging part of the iceberg!. J Crit Care.

[3] Ospina-Tascón GA, Hernandez G, Alvarez I, Calderón-Tapia LE, Manzano-Nunez R, Sánchez-Ortiz AI (2020). Effects of very early start of norepinephrine in patients with septic shock: a propensity score-based analysis. Crit Care.

[4] Hatib F, Jansen JR, Pinsky MR (1985). Peripheral vascular decoupling in porcine endotoxic shock. J Appl Physiol.

[5] O’Rourke MF (1967). Steady and pulsatile energy losses in the systemic circulation under normal conditions and in simulated arterial disease. Cardiovasc Res.

[6] Hernandez G, Messina A, Kattan E (2022). Invasive arterial pressure monitoring: much more than mean arterial pressure!. Intensive Care Med.

[7] Dellinger RP, Levy MM, Carlet JM, Bion J, Parker MM, Jaeschke R (2008). Surviving Sepsis Campaign: international guidelines for management of severe sepsis and septic shock: 2008. Crit Care Med.

[8] Dellinger RP, Levy MM, Rhodes A, Annane D, Gerlach H, Opal SM (2013). Surviving sepsis campaign: international guidelines for management of severe sepsis and septic shock: 2012. Crit Care Med.

[9] Rhodes A, Evans LE, Alhazzani W, Levy MM, Antonelli M, Ferrer R (2017). Surviving sepsis campaign: international guidelines for management of sepsis and septic shock: 2016. Intensive Care Med.

[10] Hamzaoui O, Teboul JL (2019). Importance of diastolic arterial pressure in septic shock rebuttal to comments of Dr. Magder J Crit Care.

[11] Hamzaoui O, Teboul JL (2019). Importance of diastolic arterial pressure in septic shock: PRO. J Crit Care.

[12] Ospina-Tascón GA, Teboul JL, Hernandez G, Alvarez I, Sánchez-Ortiz AI, Calderón-Tapia LE (2020). Diastolic shock index and clinical outcomes in patients with septic shock. Ann Intensive Care.

[13] Vidal-Petiot E, Ford I, Greenlaw N, Ferrari R, Fox KM, Tardif JC (2016). Cardiovascular event rates and mortality according to achieved systolic and diastolic blood pressure in patients with stable coronary artery disease: an international cohort study. Lancet.

[14] Zhu Z, Zhou M, Wei Y, Chen H (2022). Time-varying intensity of oxygen exposure is associated with mortality in critically ill patients with mechanical ventilation. Crit Care.

[15] Evans L, Rhodes A, Alhazzani W, Antonelli M, Coopersmith CM, French C (2021). Surviving sepsis campaign: international guidelines for management of sepsis and septic shock 2021. Intensive Care Med.

[16] Collet M, Huot B, Barthélémy R, Damoisel C, Payen D, Mebazaa A (2019). Influence of systemic hemodynamics on microcirculation during sepsis. J Crit Care.

[17] Sato R, Luthe SK, Nasu M (2017). Blood pressure and acute kidney injury. Crit Care.

[18] Saito S, Uchino S, Takinami M, Uezono S, Bellomo R (2016). Postoperative blood pressure deficit and acute kidney injury progression in vasopressor-dependent cardiovascular surgery patients. Crit Care.

[19] Cinel I, Kasapoglu US, Gul F, Dellinger RP (2020). The initial resuscitation of septic shock. J Crit Care.

[20] Park S, Kim DG, Suh GY, Park WJ, Jang SH, Hwang YI (2011). Significance of new-onset prolonged sinus tachycardia in a medical intensive care unit: a prospective observational study. J Crit Care.

[21] Teboul JL, Saugel B, Cecconi M, De Backer D, Hofer CK, Monnet X (2016). Less invasive hemodynamic monitoring in critically ill patients. Intensive Care Med.

[22] Benchekroune S, Karpati PC, Berton C, Nathan C, Mateo J, Chaara M (2008). Diastolic arterial blood pressure: a reliable early predictor of survival in human septic shock. J Trauma.

[23] Hamzaoui O, Scheeren TWL, Teboul JL (2017). Norepinephrine in septic shock: when and how much?. Curr Opin Crit Care.

[24] Lehman LW, Saeed M, Talmor D, Mark R, Malhotra A (2013). Methods of blood pressure measurement in the ICU. Crit Care Med.

