# Supplementary material for: The association of diastolic arterial pressure and heart rate with mortality in septic shock: a retrospective cohort study
Source: Eur J Med Res. 2022 Dec 10;27:285. doi: 10.1186/s40001-022-00930-6 (PMC9738025; doi:10.1186/s40001-022-00930-6)
Supplement: Supplementary file 2 — Additional file 2: Table S1. Baseline characteristics and outcomes of patients stratified by TWA-HR on Day 1. Table S2. Baseline characteristics and outcomes of patients stratified by TWA-DAP on Day 1. Table S3 Effects of time-dependent TWA-HR and TWA-DAP on 28-day mortality in patients with septic shock. Table S4. Effects of proportion of exposure time in tachycardia and low DAP on 28-day mortality in patients with septic shock. [file 40001_2022_930_MOESM2_ESM.docx]

**Supplementary Figure legends:**

Figure S1: Study flow diagram in the current study.

Figure S2: Time-dependent effect of TWA-HR (**a**) or TWA-DAP (**b**) on 28-day mortality.

Figure S3: Distribution of missing values for baseline variables

Figure S4: Cumulative effect of tachycardia and low DAP on 28-day mortality. (**a**) Cumulative mortality exposure to tachycardia. (**b**) Cumulative mortality exposure to tachycardia without low DAP. (**c**) Cumulative mortality exposure to tachycardia combined low DAP.

Figure S5: The direct and indirect effect of tachycardia and low DAP on 28-day mortality.

Figure S6: The interaction effect of proportion of time spent in tachycardia and low DAP on 28-day mortality.
